# Extractable and Non-Extractable Antioxidants

**DOI:** 10.3390/molecules24101933

**Published:** 2019-05-20

**Authors:** Alessandra Durazzo, Massimo Lucarini

**Affiliations:** CREA Research Centre for Food and Nutrition, Via Ardeatina 546, 00178 Rome, Italy

In addition to documented scientific interest on antioxidant phytochemicals (plant secondary metabolites) [1], the entire scientific community agrees on the importance of determination of extractable and non-extractable antioxidants [2,3,4]. In this context, the delineation and exploitation of extractable and non-extractable antioxidants in the main food groups as well as by-products [5,6,7,8,9,10,11,12,13] was the main focus of this Special Issue. This Special Issue was addressed towards the description and update of the methodological approach of antioxidant compounds in a multidisciplinary and innovative design. Conventional procedures and advanced extraction technologies, as well as analytical techniques, were considered, with particular regard to green procedures. It is worth mentioning the study of Da Silva et al. [14] on the effect of three different extraction methods—conventional (CE), ultrasound-assisted (UAE), and microwave-assisted (MAE)—on *Nectandra grandiflora* leaf extracts (NGLE) chemical yields, phenolic and flavonoid composition, physical characteristics, as well as antioxidant and antifungal properties: CE achieves the highest extraction phytochemical yield (22.16%), but with similar chemical composition to that obtained by UAE and MAE. Moreover, the authors added that CE also provided a superior thermal stability of NGLE [14].

Another example was given by Nemes et al. [15] that proposed a new process for extracting non-extractable procyanidins bound to the membrane, proteins, and fibers. Fraisse et al. [16] proposed a novel HPLC method for direct detection of nitric oxide scavengers from complex plant matrices and its application to *Aloysia triphylla* leaves.

On the other hand, Rodriguez-Jimenez et al. [17] studied physicochemical, functional, and nutraceutical properties of eggplant flours obtained by different drying methods: the drying oven flour results as a potential ingredient for the preparation of foods with functional properties, since it is rich in phenolic compounds and antioxidants. Espinosa-Páez et al. [18] reported increasing antioxidant activity and protein digestibility in *Phaseolus vulgaris* and *Avena sativa* by fermentation with the *Pleurotus ostreatus* fungus. Šic Žlabur, [19] by evaluating the possibility of using chokeberry powder as a supplement in apple juice to increase the nutritional value of the final product, showed a positive correlation between vitamin C content, total phenols, flavonoids, and anthocyanins content and antioxidant capacity in juice samples with added chokeberry powder treated with high intensity ultrasound. Durazzo et al. [20] reported the antioxidant properties of four commonly consumed popular Italian dishes our popular dishes, in terms of extractable and non-extractable antioxidants.

Particular attention was given to the studies of extractable and non-extractable antioxidants on food waste, in line with the concepts of circular economy and biorefineries. In this regard, it is worth mentioning the study of Lucarini et al. [21] on bio-based compounds from grape seeds, by giving the main lines of a biorefinery approach. Posadino et al. [22] concluded that the Naviglio extraction, as a green technology process, can be used to exploit wine waste to obtain antioxidants which can be used to produce enriched foods and nutraceuticals high in antioxidants.

The combination of emerging analytical techniques and the application of statistical methods, i.e., infrared spectroscopy, multielemental analysis, isotopic ratio mass spectrometry, and nanotechnologies coupled with chemometrics were taken into account. For instance, in the work of Kock et al. [23] on black tea samples origin discrimination using analytical investigations of secondary metabolites, antiradical scavenging activity and chemometric approach, the applied principal component analysis (PCA) and ANOVA revealed several correlations between the level of catechins in tea infusions. Anokwuru et al. [24] studied antioxidant activity and spectroscopic characteristics of extractable and non-extractable phenolics from *Terminalia sericea* Burch. ex DC.: This study demonstrated that extractable phenolics contributed more to the antioxidant activities compared to the non-extractables.

Indeed, the potential effects of extractable and non-extractable antioxidants were investigated. In this regard, the study of Zhong et al. [25] studied the effects of different degrees of procyanidin polymerization on nutrient absorption and digestive enzyme activity in mice and concluded that in the process of food production, the anti-nutritional properties of polyphenols could be minimized by reducing the degree of polymerization of proanthocyanidins. Diaconeasa et al. [26], in a study on time-dependent degradation of polyphenols from thermally-processed berries, revealed that when processed and stored in time, the bioactive compounds from berry jams are degrading, but they still exert antioxidant and antiproliferative potential.

The utilization of extractable and non-extractable antioxidants in the nutraceuticals field [3,4,27,28,29,30,31,32,33,34,35] was another focal point of this Special Issue: extracts, fractions, purified, and semi-purified substances, used alone or in combination with other ingredients as dietary supplements or functional foods. This field needs to be explored using rigorous science approaches, considering a combination of studies from different fields (nutrition, food chemistry, medicine, etc.) is increasing.

In this regard, Durazzo et al. [35] have given an updated picture of the strict interaction between main plant biologically active compounds and botanicals, by underlying actual possibilities of study approach and research strategies. Li et al. [36], by studying soluble- and insoluble-bound phenolics and antioxidant activity of two Chinese mistletoes, indicated it as source of antioxidants in human healthcare. On the other hand, Li et al. [37], by studying the inclusion complexes of daidzein with β-cyclodextrin and derivatives, showed that the antioxidant performance of the inclusion complexes was enhanced in comparison to that of the native daidzein. Moreover, Davaatseren et al. [38] evaluated the anti-inflammatory and antioxidant effects of trans-Cinnamaldehyde self-included in β-cyclodextrin complexes (CIs) in lipopolysaccharide (LPS)-treated murine RAW 264.7 macrophages: CIs may have strong anti-inflammatory and antioxidant effects, similar to those of trans-Cinnamaldehyde when used alone.

We would like to acknowledge the efforts of the authors of the publications in this Special Issue.
